# Broad host susceptibility of North American amphibian species to *Batrachochytrium salamandrivorans* suggests high invasion potential and biodiversity risk

**DOI:** 10.1038/s41467-023-38979-4

**Published:** 2023-06-05

**Authors:** Matthew J. Gray, Edward Davis Carter, Jonah Piovia-Scott, J. Patrick W. Cusaac, Anna C. Peterson, Ross D. Whetstone, Andreas Hertz, Aura Y. Muniz-Torres, Molly C. Bletz, Douglas C. Woodhams, John M. Romansic, William B. Sutton, Wesley Sheley, Allan Pessier, Catherine D. McCusker, Mark Q. Wilber, Debra L. Miller

**Affiliations:** 1 Center for Wildlife Health, School of Natural Resources, University of Tennessee, Knoxville, TN USA; 2 School of Biological Sciences, Washington State University, Vancouver, WA USA; 3 Biology Department, University of Massachusetts Boston, Boston, MA USA; 4 Smithsonian Tropical Research Institute, Ancón, Panama; 5 Department of Agricultural and Environmental Sciences, Tennessee State University, Nashville, TN USA; 6 Department of Biomedical and Diagnostic Sciences, College of Veterinary Medicine, University of Tennessee, Knoxville, Knoxville, TN USA; 7 Department of Veterinary Microbiology and Pathology, Washington State University, Pullman, WA USA

**Keywords:** Ecological epidemiology, Conservation biology

## Abstract

*Batrachochytrium salamandrivorans* (*Bsal*) is a fungal pathogen of amphibians that is emerging in Europe and could be introduced to North America through international trade or other pathways. To evaluate the risk of *Bsal* invasion to amphibian biodiversity, we performed dose-response experiments on 35 North American species from 10 families, including larvae from five species. We discovered that *Bsal* caused infection in 74% and mortality in 35% of species tested. Both salamanders and frogs became infected and developed *Bsal* chytridiomycosis. Based on our host susceptibility results, environmental suitability conditions for *Bsal*, and geographic ranges of salamanders in the United States, predicted biodiversity loss is expected to be greatest in the Appalachian Region and along the West Coast. Indices of infection and disease susceptibility suggest that North American amphibian species span a spectrum of vulnerability to *Bsal* chytridiomycosis and most amphibian communities will include an assemblage of resistant, carrier, and amplification species. Predicted salamander losses could exceed 80 species in the United States and 140 species in North America.

## Abstract (web summary)

The emerging fungal pathogen Bsal is a potential threat for amphibians. Here, the authors use data from dose-response experiments on 35 species to assess vulnerability of North American amphibians to Bsal and identify species and areas potentially at risk.

## Introduction

Wildlife trade is a burgeoning industry involving >180 nations and >$300B USD annually^1,2^, and is a common pathway for novel pathogen introduction^3,4^. The pet amphibian trade is likely responsible for the recent introduction of the emerging fungal pathogen, *Batrachochytrium salamandrivorans* (*Bsal*), from Asia into Europe^5–7^. The United States (USA) leads the global market in live amphibian imports^1^, and animal health certificates for wildlife imports are not required. *Bsal* is a necrotizing skin pathogen that is causing population declines of wild salamanders in at least four European countries^8–13^, yet has not been detected in North America^14,15^, a global hotspot for salamander biodiversity^16^. Species richness of salamanders in the USA is 5X greater than Europe^16^, and communities are composed of genera known to be susceptible to *Bsal*^5^. Risk models suggest that environmental suitability for *Bsal* is high in North America^17–19^; however, previous modeling efforts ignored geographic distributions and susceptibility of possible host species (*cf*. Moubarak et al.^20^). Species susceptibility experiments have been performed for approximately 5% of species (18/359) found in North America^5,21–24^. Those studies suggest that salamander species in the Plethodontidae (lungless salamanders) and Salamandridae (newts) may be particularly vulnerable to *Bsal* introduction. Recent results also suggest that frogs can become infected and develop the disease, *Bsal* chytridiomycosis^21^. Considering that *Bsal* can transmit rapidly between individuals and not depend on host density^25,26^, the consequences of its introduction on endemic North American amphibian assemblages could be severe.

Here, we present an extensive continental evaluation of species susceptibility to *Bsal* infection. We conducted dose-response experiments for 35 North American species from 10 amphibian families. Using estimates of infection and disease susceptibility and pathogen loads on amphibian skin, we provide evidence that North America’s amphibian communities are highly vulnerable to *Bsal* invasion, and some species are at risk of extinction should this pathogen invade. Given the high invasion risk of *Bsal* and high salamander diversity in North America, we recommend a trilateral agreement among the USA, Canada, and Mexico to reduce the risk of *Bsal* introduction and substantial biodiversity loss.

## Results and discussion

### Species susceptibility

We found that the host range for *Bsal* in North American amphibian species is broader than in European species. Whereas Martel et al.^5^ found that infection was primarily restricted to Salamandridae (newt) species, we discovered that 25 of 35 species (71%) tested became infected with *Bsal* (Supplementary Table 1), belonging to five caudate and four anuran families. Among species susceptible to infection, average incubation period was 10.5 days (SD = 11.4; Supplementary Table 1) and average duration to mortality for those species that developed fatal chytridiomycosis was 21.8 days (SD = 12; Supplementary Fig. 1), illustrating that *Bsal* epizootics could occur quickly following initial transmission events^10,11^. Furthermore, for species that developed high *Bsal* loads there was often an exponential increase in load following exposure to zoospores (Supplementary Fig. 3). The growth rate of *Bsal* on hosts also was dose-dependent, suggesting that the impacts of outbreaks could be hastened by the buildup of *Bsal* zoospores in the environment.

**Fig. 1:**
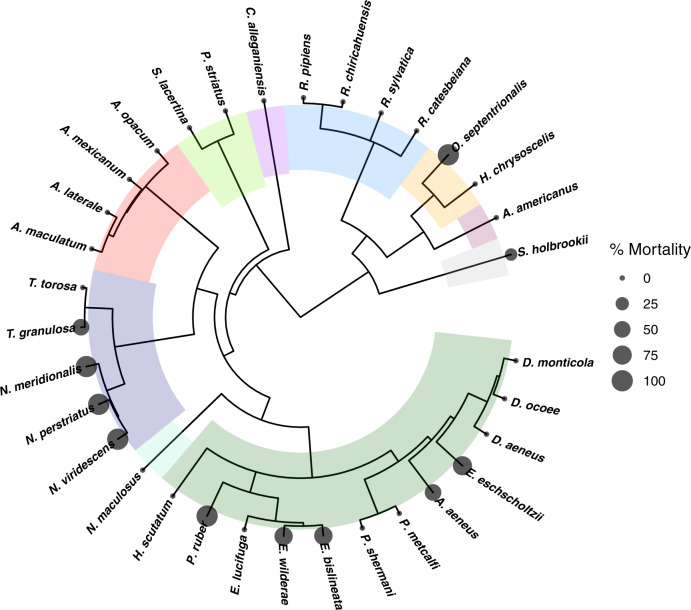
Significant phylogenetic signature was detected for *Batrachochytrium salamandrivorans* (*Bsal*)-induced mortality based on Blomberg’s K (*P* = 0.002, *K* = 0.40). Circles are scaled to indicate percent mortality experienced by individuals exposed to the highest *Bsal* zoospore dose (5 × 10^6^) (Supplementary Table 1). Colors designate different taxonomic families of amphibians. For the experiments, all species were exposed to *Bsal* zoospores inoculated in a water bath, except for *Siren lacertina*, *Cryptobranchus alleganiensis*, and *Necturus maculosus*, because these species were too large to fit in the inoculation containers. We pipetted *Bsal* on to the dorsum of these species (similar to Martel et al.^6^) at the same doses then put the animals in a water bath (see Supplementary Table 2).

We also found that *Bsal*-induced chytridiomycosis followed a phylogenetic signal (*P* = 0.009, *K* = 0.29), with the majority of mortality occurring in Plethodontidae and Salamandridae (Fig. 1; Supplementary Fig. 2), which corroborates previous findings that newts and lungless salamanders are particularly vulnerable to *Bsal*^5,22^. Of the 35 species tested, 8 (24%) were classified as having moderate to very high mortality risk (Supplementary Table 1), which included all three North American *Notophthalmus* species, four species in the family Plethodontidae, and *Osteopilus septrionalis*—a frog species that is invasive in 14 countries^21^. Three of those species (*N. meridionalis*, *N. perstriatus*, and *Aneides aeneus*) are listed as vulnerable or endangered by the IUCN^27^ (Supplementary Table 1), emphasizing the risk of *Bsal* invasion to the persistence of threatened species^10^. Five of eight (63%) frog species became infected with *Bsal* and two developed *Bsal* chytridiomycosis (Supplementary Table 1 and Supplementary Fig. 2), adding evidence that anurans can play a role in the translocation and epidemiology of *Bsal*^11,28^. We also found that salamander larvae (*Eurycea bislineata*), including some undergoing metamorphosis (*Notophthalmus viridescens*), could become infected with *Bsal* (Supplementary Table 1 and Supplementary Fig. 2), which has not been reported previously and increases the possibility that intraspecific reservoirs could influence host-pathogen dynamics, as known for other amphibian pathogens such as ranaviruses and *Bd*^29,30^. Two species (*Ambystoma mexicanum*, *Osteopilus septentrionalis*) that are commonly found in trade and not included in import bans in the USA became infected (Supplementary Table 1)^21,31^, illustrating the need for programs that facilitate healthy (clean) trade of amphibians. Our susceptibility results likely represent a first-time exposure to *Bsal*. None of the 336 animals that were randomly assigned to controls (26% of total sample size) tested positive for *Bsal*. Also, all animals were collected from the wild or from captive populations in the USA, where *Bsal* has not been detected. If true *Bsal* infection prevalence of our pre-experiment animals was 1–5%, the probability of not detecting a positive *Bsal* infection by testing 336 control animals was <3% (Supplementary Note 1). Also, none of our animals tested positive for *Bd*, nor was their histological evidence of *Bd* chytridiomycosis. Hence, our results do not represent susceptibility associated with *Bd* co-infection, which could have synergistic effects^32^.

### Geographic risk analysis

**Fig. 2:**
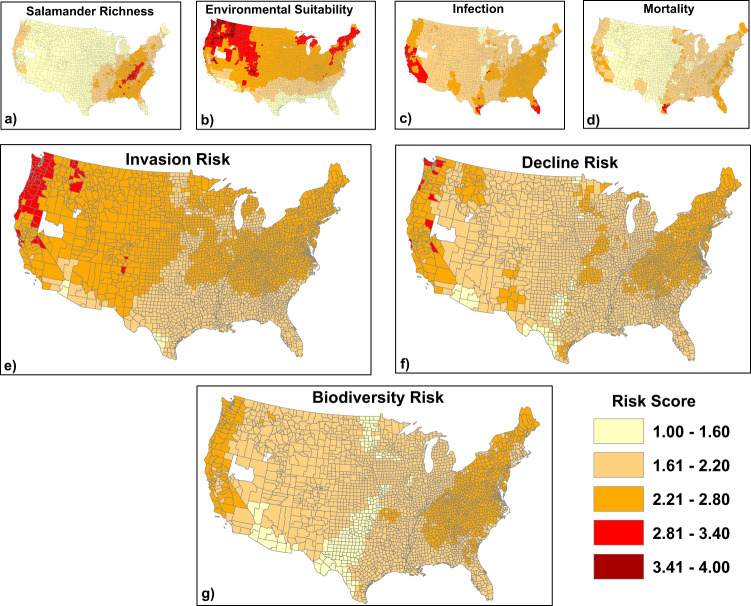
Geographic risk analyses for *Batrachochytrium salamandrivorans* (Bsal) in the United States. The top row shows salamander species richness (**a**), environmental suitability for *Bsal* (**b**) and mean predicted infection and mortality across host species at the county level (**c,** **d**). Invasion risk (**e**) was created by averaging environmental suitability (**b**) and mean predicted infection (**c**). Decline risk (**f**) is the average of **b**, **c**, and **d**; high scores indicate environmentally suitable areas where the host community includes species that are readily infected and experience *Bsal*-related mortality. Biodiversity risk (**g**) due to *Bsal* also takes species richness into consideration (i.e., is the average of **a**–**d**), and indicates where salamander diversity will be impacted most. Darker shades indicate greater risk.

Inasmuch as likelihood of infection and mortality by *Bsal* appear to be phylogenetically conserved (Martel et al.^5^, Fig. 1), we mapped predicted risk of *Bsal* invasion and biodiversity loss for salamander communities in the USA (Fig. 2). Predicted invasion risk was moderate to high throughout most of the USA (Fig. 2e), with the northwestern USA and portions of Colorado and New Mexico having greatest risk, likely due to high environmental suitability for *Bsal* and the presence of multiple host species in resident communities. We combined invasion risk with estimated mortality risk and species richness to identify the West Coast and Appalachian Mountains as regions where biodiversity loss is expected to be most severe (Fig. 2g). Previous risk assessments corroborate these findings, although in general, they assigned greater risk to the southeastern USA and near ports-of-entry due to their model assumptions^17,18^. Our results indicate that although salamander species richness is greatest in the southeastern USA, amphibian communities in this region consist of species with lower susceptibility or inhabit locations where temperatures exceed the thermal optimum for *Bsal* infection. Factoring temperature-dependent susceptibility into likelihood of *Bsal* infection^11,33–35^ shifted risk north and to higher elevations in our analyses compared to previous assessments, as suggested by Carter et al.^35^. Moubarak et al.^20^ recently used machine learning and ecological niche modeling to predict species susceptibility to *Bsal* and invasion risk in the USA. Their models also predicted *Bsal* invasion farther south than our assessment, possibly due to the limited data on host susceptibility to *Bsal* available for use in their analyses. In contrast to several previous analyses (e.g., Yap et al.^17^, Richgels et al.^18^, Grear et al.^36^), we excluded ports-of-entry or pet store locations from our risk assessment because they could be misleading, as they probably do not represent likely point-source locations for future *Bsal* introductions. Unwanted pet amphibians and release by consumers into the wild represents the greatest risk of introduction, which could occur anywhere. For our assessment, we assumed no spatial constraints on location of *Bsal* introduction, hence our predictions were influenced only by environmental conditions, amphibian distributions, and their susceptibility to *Bsal* infection and death. Greater understanding of the movement of international and domestic amphibians through trade networks and likely locations for spillover is needed to appropriately incorporate business and consumer behaviors into *Bsal* risk assessments. Our assessment represents the possible consequences of a point-source introduction of *Bsal* and does not consider amphibian dispersal or landscape features (such as topography or urban development) that could subsequently impact spatial epidemiology. Research in Europe suggests that positive *Bsal* sites with population declines can be located near uninfected sites, especially if barriers to amphibian dispersal exist^37^. Lastly, although our predictions consider the environmental suitability of *Bsal*, they do not consider fine-scale, microhabitat conditions nor predictions associated with climate change. Future research should evaluate how changes in microhabitat and climate influence *Bsal*-host interactions.

### Possible epidemiological roles

**Fig. 3:**
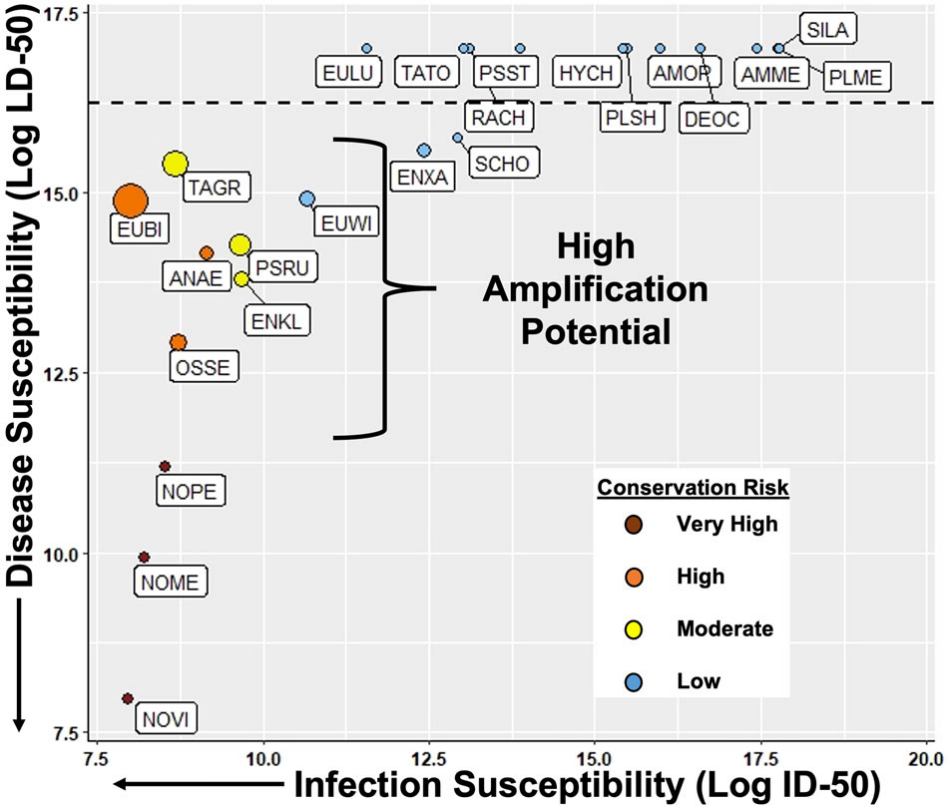
Host susceptibility and amplification potential for amphibian species that became infected with *Batrachochytrium salamandrivorans* (*Bsal*). Host susceptibility is graphically represented by plotting infection susceptibility (measured as infectious dose [ID]−50) against disease susceptibility (measured as lethal dose [LD]−50), hence species with low ID-50 and LD-50 values (i.e., near graph origin) were most susceptible and those in the upper right quadrant were least susceptible. Arrows on the axes indicate direction of increasing infection and disease susceptibility. Colors correspond to their mortality risk (also referred to as Conservation Risk, Supplementary Table 1), which was calculated as the product of ID-50 and LD-50 values, with categories designated using 25^th^ percentile quartiles. Amplification potential was estimated as the ratio of LD-50 and ID-50 values multiplied by transmission potential (i.e., average *Batrachochytrium salamandrivorans* [*Bsal*] load on skin swabs) for each species (Supplementary Table 1). Larger circles indicate greater amplification potential. The species above the dotted line were considered to be potential carriers, because they became infected with *Bsal* but no disease-induced mortality occurred (i.e., LD-50 was not estimable). Carrier species above the dotted and toward the left side of the graph likely have greater potential to transmit *Bsal* because they become infected at lower zoospore doses. Resistant species are not plotted (Supplementary Table 1). AMME *Ambystoma mexicanum*, AMOP *Ambystoma opacum*, ANAE *Aneides aeneus*, DEOC *Desmognathus ocoee*, ENKL *Ensatina eschscholtzii klauberi*, ENXA *Ensatina eschscholtzii xanthoptica*, EUBI *Eurycea bislineata*, EULU *Eurycea lucifuga*, EUWI *Eurycea wilderae*, HYCH *Hyla chrysoscelis*, NOME *Notophthalmus meridionalis*, NOPE *Notophthalmus perstriatus* NOVI *Notophthalmus viridescens*, OSSE=*Osteopilus septentrionalis*, PLME *Plethodon metcalfi*, PLSH *Plethodon shermani*, PSST *Pseudobranchus striatus*, PSRU *Pseudotriton ruber*, RACH *Rana chiricahuensis*, SCHO *Scaphiopus holbrookii*, SILA *Siren lacertina*, TAGR *Taricha granulosa*, and TATO *Taricha torosa*. For the experiments, all species were exposed to *Bsal* zoospores inoculated in a water bath, except for SILA because this species was too large to fit in the inoculation containers. We pipetted *Bsal* on to the dorsum of SILA (similar to Martel et al.^6^) at the same doses then put the animal in a water bath (see Supplementary Table 2).

We estimated median infectious and lethal doses (ID-50 and LD-50 values, respectively) for species with sufficient infection and mortality data, calculated the quotient of LD-50 and ID-50 values, and multiplied this quotient by mean *Bsal* load for a novel estimate of amplification potential (Supplementary Table 1). In the context of our study, species with high amplification potential may contribute disproportionally to transmission events, because they are easy to infect, less likely to die quickly from infection (which is expected to increase the duration of infection), and likely to be more infectious due to greater pathogen loads on their skin. Seven species (20%) were classified as having high amplification potential—one of which is an invasive frog species, *O. septentrionalis* (Fig. 3; Supplementary Table 1). The 13 additional species (37%) that were susceptible to infection but did not die from *Bsal* chytridiomycosis are likely carriers (Fig. 3); some of those species (e.g., *Hyla chrysoscelis*) have widespread distribution and use a range of habitats, including urban environments. Taken together, these results illustrate there is tremendous potential for amphibian communities in North America to be composed of carrier species that serve as reservoirs, amplification species that contribute disproportionately to *Bsal* transmission^38^, and chytridiomycosis susceptible species that are at high risk of population decline and extirpation. Future studies need to evaluate whether species composition translates to community-level transmission dynamics and predicted population declines, considering various factors impact interspecific transmission. Our experimental data could be used to parameterize multi-species epidemiological models as an initial step to make community-level inferences.

## Conclusions

Our results confirm high invasion potential for *Bsal* and extinction risk for salamander species in North America^18,19^. A large number of susceptible host species and the capability of hosts to function in multiple epidemiological roles (e.g., carrier and amplification species) provide the perfect combination for high community-level invasion potential, transmission, and persistence^38^. Some species also exist at high densities in the wild and have excellent dispersal ability (e.g., *N. viridescens*, *T*. *granulosa*^39^), increasing the likelihood of *Bsal* transmission^25^ and overland spread. Possible infection of frogs and larval, juvenile, and adult stages of salamanders increase opportunities for *Bsal* persistence by providing multiple potential reservoirs. Our maps explicitly consider the susceptibility of species present to *Bsal*; thus, they can be used to respond strategically to an outbreak or proactively target surveillance in high-risk locations. Our maps should not be used to predict actual transmission dynamics within an amphibian community. Considering the high invasion risk of *Bsal* and threat to biodiversity in North America that our study and others have reported^17–20,36^, we recommend that the USA, Canada, and Mexico develop a trilateral agreement to support pathogen-free trade of amphibians. If our estimates of susceptibility to *Bsal* infection (76%) and chytridiomycosis (39%; Supplementary Table 1) for salamander taxa are representative, we expect that, among salamanders, at least 270 species are suitable hosts and >140 species could be at risk of population declines or extinction in North America (ca. 360 species total^16^) if *Bsal* invasion occurs. A trilateral agreement should consider the interests of and be in partnership with the amphibian trade industry. Recent socioeconomic research suggests that U.S. pet amphibian businesses are in support of a healthy trade program, where sold amphibians are verified to be negative for *Bsal*, *Bd*, and ranavirus^40^. A certification program that facilitates animal testing and implementation of prudent biosecurity practices in businesses is needed. The program also should provide resources for training business personnel and compensating businesses for costs associated with pathogen containment following detection and treatment or humane euthanasia of infected individuals. Healthy trade programs exist for agricultural and domesticated animals^41^, but are uncommon for wildlife.

## Methods

### Experiments

Amphibians used in our experiments were either collected from the wild or provided from captive colonies (Supplementary Table 2). Selection of species was based on availability and maximizing phylogenetic coverage, especially for salamanders. When possible, we tested larvae because their role in *Bsal* epidemiology is largely unknown^11,42^. For this study, we defined larvae as the pre-metamorphosis stage, juveniles as post-metamorphosis but <1 year, and adults as >1 year post-metamorphosis. The exception was *N. viridescens* juveniles (efts), which can be identified phenotypically (aposematic coloration) and remain in this stage for up to eight years^43^. Sex could not be distinguished for most species, hence was not used as a factor in the experimental design. Data on susceptibility to *Bsal* for four species of the 35 species (*Pseudotriton ruber*, *Eurycea wilderae*, *Eurycea* *lucifuga*, and *O. septentrionalis*) were reported previously^21,22^.

Animals were shipped overnight to the University of Tennessee (29 species), University of Massachusetts-Boston (5 species), or Washington State University-Vancouver (1 species) in individual sterile plastic containers with moistened paper towels (if a terrestrial species) or in water (if an aquatic species). Although experiments were conducted at three institutions and not simultaneously (Supplementary Table 2), laboratory procedures and diagnostic assays followed the same methods. Upon arrival, animals were tested for *Bd* infections using qPCR; all individuals used in the experiments were negative for *Bd* prior to experimental exposure. Also, no histological evidence of co-infection with *Bd* existed; thus, our results represent species responses to *Bsal* exposure only. It also is highly unlikely that our species were exposed to *Bsal* prior to our experiments, because *Bsal* has not been detected in North America and none of the animals randomly assigned to controls (*n* = 336, 26% of total sample size) ever tested positive for *Bsal*.

In the laboratory, terrestrial amphibians were held in 710-mL plastic containers with a moist paper towel and plastic, opaque cover object to reduce stress. Fully aquatic amphibians or larvae were held in either 20-L glass aquaria (large aquatic [e.g., *Siren*] or stream-dwelling [*Cryptobranchus*] species) or in 1–3-L tubs containing 500 mL of dechlorinated water and a plastic cover object, depending on body size (Supplementary Table 2). Animals were fed a diet of either brine shrimp, alfalfa pellets, blood worms, bean beetles, fruit flies or crickets every three days depending on their age class, body size and whether they were aquatic or terrestrial. Animals were acclimated in the laboratory ca. 2 weeks before experiments began. Containers were maintained between 20–22 °C until one week prior to *Bsal* exposure, after which they were placed in Conviron® environmental chambers (Winnipeg, Canada) or temperature-controlled rooms. The temperature was decreased by 3 °C per day until reaching the target temperature of 15 °C, which has been the standard for *Bsal* experiments testing host susceptibility^5,22^ and corresponds with optimum *Bsal* growth in vitro^8^. The ambient light:dark cycle prior to and during experiments was 12:12 h, respectively.

The methodology and design for experimental exposures followed Carter et al.^22^, and are summarized in the Supplementary Table 2. The *Bsal* isolate (AMFP13/1) was provided by Ghent University from the index case for *Bsal* outbreaks in the Netherlands (Martel et al.^8^). Number of isolate passages was between 10 – 20 among species and laboratories, hence considered low passage^44^. Amphibians were exposed to 1–4 *Bsal* doses (5 × 10^3–^^6^ *Bsal* zoospores in 10 mL of water) in a 100-mL conical container that could accommodate the body size of the amphibian for 24 h then returned to their housing container. The 10-mL water bath was sufficient liquid to partially cover feet and venter of post-metamorphic amphibians and submerge larvae. For three large aquatic species that could not fit in inoculum containers (*Siren lacertina*, *Cryptobranchus alleganiensis*, *Necturus maculosus*), *Bsal* was pipetted on to the dorsum similar to Martel et al.^5^, and they were placed in 1-L containers with 500 mL of water for 24 hrs. Kumar et al.^44^ reported that observed mortality from water-bath exposure to *Bsal* can differ from direct pipette exposure, hence we caution direct interpretation of susceptibility between species with different exposure methods. For all species, we included control animals that were treated identically to exposed animals (Supplementary Table 2). Inasmuch as captivity can induce stress in amphibians^45^, our estimates of susceptibility are most appropriate for relative comparisons.

Non-larval animals were placed in new housing containers with fresh materials every three days. Larval experiments were conducted on an aquatics rack system (Aquaneering Inc., San Diego, CA) that performed automated water changes every 12–24 h. After exposure, animals were monitored twice daily. If an individual lost righting reflex or mobility (a gross sign of *Bsal* chytridiomycosis^46^), it was humanely euthanized using benzocaine hydrochloride or tricaine methanesulfonate, and recorded as a mortality event for analyses. Animals were swabbed every 6–7 days beginning four days post-exposure to test for *Bsal* infection and estimate pathogen load. The ventrum and each foot was swabbed 10X each, as typical for *Bd* and *Bsal* sampling (Blooi et al.^47^). Experiments lasted 45–60 days, which is adequate time for *Bsal* chytridiomycosis to develop^5^. All species were tested at the University of Tennessee except for *Taricha torosa* (Washington State University), *Ambystoma maculatum* (University of Massachusetts-Boston), and the larval species listed in Supplementary Table 2 (University of Massachusetts-Boston). Methods were the same among institutions and the same *Bsal* isolate was used in all experiments.

Genomic DNA was extracted from swabs using a Qiagen DNeasy blood and tissue kit (Hilden, Germany), and quantitative-PCR (qPCR) assays were used to detect *Bsal* and *Bd* DNA and estimate load (as described in Carter et al.^22^). We considered an individual infected if two consecutive swabs or three or more swabs during the entire experiment were qPCR-positive for *Bsal* DNA. Incubation period was estimated by averaging the number of days it took each individual to test positive for *Bsal* DNA using qPCR for each species. Gross lesions were noted during necropsy and reported; however, we used histopathology to verify *Bsal* chytridiomycosis as per the case definition for all species that were qPCR-positive and experienced mortality (Thomas et al.^46^; Supplementary Fig. 2). For histopathology, at least five formalin-fixed individuals per species were cross-sectioned. The cross-sections were placed into tissue cassettes, routinely processed, embedded in paraffin blocks, sectioned at 3–5 µm. Sections were placed onto glass slides, stained with hematoxylin and eosin, cover-slipped and examined by light microscopy for evidence of skin infection by *Bsal*. The veterinary pathologists (DLM, AP) that processed the samples were not blinded to sample identity, as the goal was to find definitive evidence of *Bsal* chytridiomycosis. All experiments followed Biosafety Level 2 containment strategies.

### Ethics statement

All procedures followed husbandry and euthanasia recommendations provided by the American Veterinary Medical Association and the Association of Zoos and Aquariums. All animal procedures were approved under Institutional Animal Care and Use Committee (IACUC) protocols 2395, 2014003, and 4749 at the University of Tennessee, University of Massachusetts-Boston and Washington State University-Vancouver, respectively. IACUC approval for the *Atelopus zeteki* experiment was obtained from the Maryland Zoo.

### *Bsal* growth

We compared *Bsal* growth rates among zoospore exposure doses using a zero-inflated negative binomial (ZINB) mixed model. For these analyses, we only included the 12 species for which at least one of the zoospore doses reached a mean infection intensity > 10^3^ copies uL^−1^ (means were calculated using log_10_ transformed data) in at least one of the weeks of the experiment, which was necessary to parametrize the model. If a given species met this data requirement in one dose, we included all doses for that species. In order to characterize initial growth rates, we used data from the exponential portion of the pathogen growth curve (i.e., the period after exposure when log[*Bsal* load] was linear). We defined this portion of the pathogen growth curve as the period from the beginning of each experiment until the first week in which the mean pathogen load was 90% or more of the maximum weekly mean pathogen load for that dose during the experiment. If the maximum weekly mean pathogen load never exceeded 100 copies uL^−1^ for a particular dose, we used all weeks of data. This decision rule ensured that the model (which assumes exponential growth) targeted pathogen growth when this assumption was most reasonable.

The fixed effects for both the negative binomial and the zero-inflated components of the model were day after exposure (as a continuous predictor), dose, and their interaction. The random effects were species and individual animal (which was nested within species). For species, the random effects included both a random intercept and a random slope. All random effects were included in both the negative binomial and the zero-inflation components of the model. The response variable (*Bsal* copies µL^−1^) was converted to integer values prior to analysis in accordance with the use of a negative binomial error distribution. Models were fit using the glmmTMB package^48^ in R; conformity with model assumptions was evaluated using the DHARMa package^49^. We evaluated the significance of the day of exposure*dose interaction using a likelihood ratio test in which the full model was compared to a model where the interaction term was removed from both the zero-inflated and negative binomial parts of the model. Parameters from the fitted model are presented in Supplementary Table 3. Post-hoc tests evaluating the differences in slopes between doses were conducted separately for the zero-inflated and negative binomial portions of the model using *t*-tests with Tukey adjustments for multiple comparisons (Supplementary Table 4); these post-hoc tests were performed using functions in the emmeans package^50^.

### Analyses of susceptibility and amplification potential

For species or age classes that became infected or died, we estimated median infectious and lethal doses (ID-50 and LD-50 values, respectively) using log-probit analyses in the MASS package in R^51,52^. For these analyses, the response variable was percent infection or mortality per dose and the categorical explanatory variable was zoospore dose. Given ID-50 is an estimate of infection likelihood^53^, we considered ID-50 as a measure of infection susceptibility. Similarly, we considered LD-50 as a measure of disease susceptibility. Because the ID-50 and LD-50 represent two risk factors of host survival, we calculated their product as a measure of overall host susceptibility and mortality risk^54^. We calculated quartiles for this quantity and ranked host species as: 0–25% = very high, 26–50% = high, 51–75% = moderate, and 76–100% = low (Fig. 3; Supplementary Table 1). Hence, species with low ID- and LD-50 products were deemed to be most susceptible and have greatest mortality risk, while those with high ID- and LD-50 products were classified as lower mortality risk. We also deemed all species that became infected with *Bsal*, but did not die, as low mortality risk (Fig. 3; Supplementary Table 1). The species that did not become infected were classified as resistant (Supplementary Table 1).

We took a unique approach to estimating host amplification potential (Paull et al.^38^) by taking the quotient of the LD-50 and ID-50, and multiplying by the average load of *Bsal* detected on the skin during the experiments. Species with high quotients became infected at low exposure doses, but only developed fatal *Bsal* chytridiomycosis at high exposure doses, hence increasing their potential to remain infected in the environment for longer duration. We assumed that greater *Bsal* loads on amphibian skin could lead to greater transmission events either through direct contact or greater zoospore shedding rates (as seen with *Bd*^55,56^). Hence, our estimate of amplification potential considered host susceptibility (i.e., ID-50 and LD-50 estimates) and host competence (i.e., pathogen load) as suggested by Paull et al.^38^. Our estimates of amplification potential did not consider host contact rates explicitly (Paull et al.^38^), because those data do not exist for our species. Although all species that become infected with *Bsal* have the potential to be carriers, we considered species that became infected (i.e., estimable ID-50) and did not develop chytridiomycosis (i.e., inestimable LD-50) as having potential to transmit *Bsal* (Fig. 3).

To determine whether *Bsal* susceptibility followed a phylogenetic signal, we constructed a phylogenetic tree for the adult amphibians tested using TimeTree^57^, and estimated Blomberg’s K^58^ using the “Kcalc” function from the “picante”^59^ package in R (version 4.0.2)^52^. We conducted separate analyses for percent infection and mortality in the highest exposure dose (5 × 10^6^). We did not evaluate if amplification potential followed a phylogenic signal because of the limited number of species where both the LD-50 and ID-50 concentrations were estimable (Supplementary Table 1). We used separate phylogenetic reconstructions to estimate percent infection and mortality for 144 untested salamander species that had known geographic ranges within the contiguous USA^27^. Thus, predicted species infection and mortality due to *Bsal* was weighted based on phylogenetic relatedness. Specifically, we used the “phyEstimate” function from the “picante”^59^ package for the 24 salamander species with data from the adult experiments to create the ancestral trait reconstruction by assuming a phylogenetic signal for *Bsal* infection and mortality, which our data suggested (see Fig. 1, for example)^59^. Of the 175 salamander species found in the USA, seven species did not have phylogenetic information listed and were removed from the analysis. These species included *Ambystoma annulatum, A. bishopi, A. mavortium*, *Eurycea robusta*, *Gyrinophilus subterraneus, Plethodon ainsworthi*, and *Taricha sierra*. Although the geographic range of *A. mexicanum* does not extend into the USA, we included this species in the phylogenetic analysis because it is widely distributed across the USA as a popular pet and laboratory animal, and some invasive populations exist. Because experimental animals were obtained from single source populations, inferences were drawn on higher taxonomic levels rather than at the species level.

### Geographic risk analyses

We combined species-level estimates of infection and mortality from the phylogenetic analyses with information on species distributions and environmental suitability for *Bsal* to generate geographic projections of *Bsal* risk. To estimate the geographic range of each salamander species, we extracted species polygons for the 168 salamander species with known phylogenetic relationships from the IUCN species distribution database (iucnredlist.org)^27^. We used the geographic distribution polygons to generate county-level occurrences for each species using the clip function found in the ArcMap (v.10.7) analysis toolbox. We used the resulting data to create county-level averages of percent infection and mortality across species, and to calculate salamander richness. We used methods similar to those described by Richgels et al.^18^ to create three *Bsal* risk scores: invasion, mortality and biodiversity risk. Each county-level value (i.e., average predicted infection among species, average predicted mortality among species, and salamander richness) was linearly transformed to a score that ranged from 1 to 4, with 4 indicating greatest risk.

We also created an environmental suitability score for each county based on the average annual and warmest month temperatures (similar to Richgels et al.^18^), using temperature data obtained from PRISM climate normals^60^. Our temperature thresholds were determined by the temperature-dependent infection and survival of *Notophthalmus viridescens* described in Carter et al.^35^, who found that all 5 × 10^6^ exposed *N. viridescens* became infected and experienced 100% mortality at 6 and 14 °C, whereas individuals at 22 °C did not become infected. Therefore, we scaled annual ambient and warmest month temperatures = 4 (i.e., greatest environmental suitability) if estimates for that county were between 6 and 14 °C. Temperatures exceeding 14 °C were scaled uniformly from four to one as they approached 22 °C, with all temperatures exceeding 22 °C receiving a score of one. Similarly, we scaled environmental suitability from four to one for temperatures lower than 6 °C. If the assigned ranks for annual ambient and warmest month temperatures were different in a county (e.g., 3 = annual, 4 = warmest), we averaged them for the final assigned environmental suitability score (i.e., 3.5 in this example). We subsequently produced a *Bsal* environmental suitability map using the environmental suitability score generated for each county.

Using the infection, mortality, and biodiversity risk scores generated for each county (i.e., mean percent infection, mean percent mortality, species richness) and environmental suitability, we created several aggregate maps that estimated different aspects of *Bsal* risk. We created a *Bsal* invasion risk map, which was the average of the infection and environmental suitability scores (defined above) generated for each county. We averaged the mortality, infection, and environmental suitability scores to produce the decline risk score. Incorporation of the mortality score into this calculation provided a measure of amphibian population declines that may result, if areas with high environmental suitability and highly susceptible hosts are invaded by *Bsal*. Lastly, we created a biodiversity risk score for each county. The biodiversity risk score was estimated by averaging all risk scores used to determine decline risk, in addition to the salamander richness score for each county. High biodiversity risk scores indicate areas where the environment is suitable for *Bsal*, and the greatest decrease in salamander diversity is expected because the host community is composed of species that can be readily infected and develop *Bsal* chytridiomycosis.

### Reporting summary

Further information on research design is available in the Nature Portfolio Reporting Summary linked to this article.

## Supplementary information

Supplementary Information File

Reporting Summary

## Data Availability

Summarized data generated in this study are provided in the main text or the supplementary materials. Raw data used for analyses are provided in the public repository, TRACE (10.7290/pJ8IWH7DuE).

## Code availability

The code for statistical analyses used in this study is available in TRACE (10.7290/pJ8IWH7DuE).

## References

[1] Can ÖE, D’Cruze N, Macdonald DW (2019). Dealing in deadly pathogens: taking stock of the legal trade in live wildlife and potential risks to human health. Glob. Ecol. Conserv..

[2] Smith KM (2017). Summarizing US wildlife trade with an eye toward assessing the risk of infectious disease introduction. EcoHealth.

[3] Karesh WB (2012). Ecology of zoonoses: natural and unnatural histories. Lancet.

[4] Springborn MR (2015). Integrating invasion and disease in the risk assessment of live bird trade. Divers. Distrib..

[5] Martel A (2014). Recent introduction of a chytrid fungus endangers Western Palearctic salamanders. Science.

[6] Laking AE, Ngo HN, Pasmans F, Martel A, Nguyen TT (2017). *Batrachochytrium salamandrivorans* is the predominant chytrid fungus in *Vietnamese salamanders*. Sci. Rep..

[7] Fitzpatrick LD, Pasmans F, Martel A, Cunningham AA (2018). Epidemiological tracing of *Batrachochytrium salamandrivorans* identifies widespread infection and associated mortalities in private amphibian collections. Sci. Rep..

[8] Martel A (2013). *Batrachochytrium salamandrivorans* sp nov causes lethal chytridiomycosis in amphibians. Proc. Natl Acad. Sci. USA.

[9] Spitzen-van der Sluijs A (2016). Expanding distribution of lethal amphibian fungus *Batrachochytrium salamandrivorans* in Europe. Emerg. Infect. Dis..

[10] Martel, A. et al. Integral chain management of wildlife diseases. *Conserv. Lett.* e12707. 10.1111/conl.12707 (2020).

[11] Stegen G (2017). Drivers of salamander extirpation mediated by *Batrachochytrium salamandrivorans*. Nature.

[12] Lötters S, Veith M, Wagner N, Martel A, Pasmans F (2020). *Bsal-*driven salamander mortality pre-dates the European index outbreak. Salamandra.

[13] Schmeller DS, Utzel R, Pasmans F, Martel A (2020). *Batrachochytrium salamandrivorans* kills alpine newts (*Ichthyosaura alpestris*) in southernmost Germany. Salamandra.

[14] Waddle JH (2020). *Batrachochytrium salamandrivorans* (*Bsal*) not detected in an intensive survey of wild North American amphibians. Sci. Rep..

[15] Klocke B (2017). *Batrachochytrium salamandrivorans* not detected in U.S. survey of pet salamanders. Sci. Rep..

[16] AmphibiaWeb. https://amphibiaweb.org (2020).

[17] Yap TA, Koo MS, Ambrose RF, Wake DB, Vredenburg VT (2015). Averting a North American biodiversity crisis. Science.

[18] Richgels, K. L. D., Russell, R. E., Adams, M. J., White, C. L. & Grant, E. H. C. Spatial variation in risk and consequence of *Batrachochytrium salamandrivorans* introduction in the USA. *R. Soc. Open Sci.* **3**, 10.1098/rsos.150616 (2016). 10.1098/rsos.150616 PMC4785982 26998331

[19] Basanta MD, Rebollar EA, Parra-Olea G (2019). Potential risk of *Batrachochytrium salamandrivorans* in Mexico. PLoS ONE.

[20] Moubarak M, Fischhoff IR, Han BA, Castellanos AA (2022). A spatially explicit risk assessment of salamander populations to *Batrachochytrium salamandrivorans* in the United States. Divers. Distrib..

[21] Towe, A. E. et al. *Batrachochytrium salamandrivorans* can devour more than salamanders. *J. Wildl. Dis.*10.7589/jwd-d-20-00214 (2021). 10.7589/JWD-D-20-00214 34516643

[22] Carter ED (2019). Conservation risk of *Batrachochytrium salamandrivorans* to endemic lungless salamanders. Conserv. Lett..

[23] Friday B, Holzheuser C, Lips KR, Longo AV (2020). Preparing for invasion: assessing risk of infection by chytrid fungi in southeastern plethodontid salamanders. J. Exp. Zool. Part A Ecol. Integr. Physiol..

[24] Wilber MQ, Carter ED, Gray MJ, Briggs CJ (2021). Putative resistance and tolerance mechanisms have little impact on disease progression for an emerging salamander pathogen. Funct. Ecol..

[25] Malagon DA (2020). Host density and habitat structure influence host contact rates and *Batrachochytrium salamandrivorans* transmission. Sci. Rep..

[26] Tompros, A. et al. Frequency-dependent transmission of *Batrachochytrium salamandrivorans* in eastern newts. *Transbound. Emerg. Dis.*10.1111/tbed.14043 (2021). 10.1111/tbed.14043 PMC9290712 33617686

[27] IUCN. Vol. Version 2019-3 (2020).

[28] Nguyen TT, Nguyen ThV, Ziegler T, Pasmans F, Martel A (2017). Trade in wild anurans vectors the urodelan pathogen *Batrachochytrium salamandrivorans* into Europe. Amphib. Reptil..

[29] Brunner J, Schock D, Davidson E, Collins J (2004). Intraspecific reservoirs: complex life history and the persistence of a lethal ranavirus. Ecology.

[30] Rachowicz LJ, Vredenburg VT (2004). Transmission of *Batrachochytrium dendrobatidis* within and between amphibian life stages. Dis. Aquat. Org..

[31] USFWS. Injurious Wildlife Species; Listing Salamanders Due to Risk of Salamander Chytrid Fungus. Vol. 81 (ed US Fish and Wildlife Service) 1534–1556 (Federal Register, 2016).

[32] Longo, A., Fleischer, R. C. & Lips, K. Double trouble: co-infections of chytrid fungi will severely impact widely distributed newts. *Biol. Invasions* **21**, 10.1007/s10530-019-01973-3 (2019).

[33] Beukema W (2018). Environmental context and differences between native and invasive observed niches of *Batrachochytrium salamandrivorans* affect invasion risk assessments in the Western Palaearctic. Divers. Distrib..

[34] Blooi M (2015). Treatment of urodelans based on temperature dependent infection dynamics of *Batrachochytrium salamandrivorans*. Sci. Rep..

[35] Carter ED (2021). Winter is coming–Temperature affects immune defenses and susceptibility to Batrachochytrium salamandrivorans. PLoS Pathog..

[36] Grear DA, Mosher BA, Richgels KLD, Grant EHC (2021). Evaluation of regulatory action and surveillance as preventiverisk-mitigation to an emerging global amphibian pathogen *Batrachochytrium salamandrivorans* (Bsal). Biol. Conserv..

[37] Spitzen-van der Sluijs A (2018). Post-epizootic salamander persistence in a disease-free refugium suggests poor dispersal ability of Batrachochytrium salamandrivorans. Sci. Rep..

[38] Paull SH (2012). From superspreaders to disease hotspots: linking transmission across hosts and space. Front. Ecol. Environ..

[39] Petranka, J. W. *Salamanders of the United States and Canada* (Smithsonian Books, 2010).

[40] Cavasos, K. et al. Exploring business stakeholder engagement in sustainable business practices: Evidence from the US pet amphibian industry. *Bus Strategy Environ.*10.1002/bse.3455 (2023).

[41] WOAH. *World Organisation for Animal Health.* https://www.woah.org/en/home/ (2023).

[42] Van Rooij P, Martel A, Haesebrouck F, Pasmans F (2015). Amphibian chytridiomycosis: a review with focus on fungus-host interactions. Vet. Res..

[43] Gill DE (1978). The metapopulation ecology of the red-spotted newt, *Notopht**halmus viridescens* (Rafinesque). Ecol. Monogr..

[44] Kumar, R. et al. Experimental methodologies can affect pathogenicity of *Batrachochytrium salamandrivorans* infections. *PLoS ONE* **15**, e0235370 (2020). 10.1371/journal.pone.0235370 PMC7485798 32915779

[45] de Assis VR, Titon SCM, Barsotti AMG, Titon B, Gomes FR (2005). Effects of acute restraint stress, prolonged captivitystress and transdermal corticosterone application on immunocompetence and plasma levels of corticosterone on the Cururu toad (*Rhinella icterica*). PLoS ONE.

[46] Thomas V (2018). Recommendations on diagnostic tools for *Batrachochytrium salamandrivorans*. Transbound. Emerg. Dis..

[47] Blooi M (2013). Duplex real-time PCR for rapid simultaneous detection of *Batrachochytrium dendrobatidis* and Batrachochytrium salamandrivorans in Amphibian samples. J. Clin. Microbiol..

[48] Brooks M (2017). glmmTMB balances speed and flexibility among packages for zero-inflated generalized linear mixed modeling. R. J..

[49] Hartig, F. DHARMa: Residual Diagnostics for Hierarchical (multilevel/mixed) Regression Models. R package version 0.3.3.0. https://CRAN.R-project.org/package=DHARMa (2020).

[50] Length, R. et al. emmeans: Estimated Marginal Means, Aka Least-squares Means. 1.4.8 https://CRAN.R-project.org/package=emmeans (2020).

[51] Venables, W. N., Ripley, B. D. & Isbn, S. Statistics Complements to Modern Applied Statistics with S Fourth edition by. https://www.r-project.org/ (2002).

[52] R Core Team. *R: A Language and Environment for Statistical Computing.* https://www.R-project.org (2021).

[53] Roederer M (2015). Parsimonious determination of the optimal infectious dose of a pathogen for nonhuman primate models. PLOS Pathog..

[54] Vose, D. *Risk Analysis: A Quantitative Guide* (John Wiley & Sons, 2008).

[55] Ohmer MEB, Cramp RL, White CR, Franklin CE (2015). Skin sloughing rate increases with chytrid fungus infection load in a susceptible amphibian. Funct. Ecol..

[56] Maguire C (2016). Dead or alive? Viability of chytrid zoospores shed from live amphibian hosts. Dis. Aquat. Org..

[57] Hedges SB, Dudley J, Kumar S (2006). TimeTree: a public knowledge-base of divergence times among organisms. Bioinformatics.

[58] Blomberg SP, Garland T, Ives AR (2003). Testing for phylogenetic signal in comparative data: behavioral traits are more labile. Evolution.

[59] Kembel SW (2010). Picante: R tools for integrating phylogenies and ecology. Bioinformatics.

[60] PRISM Climate Group. 30-year Normals. (Oregon State University, Corvallis, OR, 2020).

