# Supplementary material for: Broad host susceptibility of North American amphibian species to Batrachochytrium salamandrivorans suggests high invasion potential and biodiversity risk
Source: Nat Commun. 2023 Jun 5;14:3270. doi: 10.1038/s41467-023-38979-4 (PMC10241899; doi:10.1038/s41467-023-38979-4)
Supplement: Supplementary file 1 — Supplementary Information File [file 41467_2023_38979_MOESM1_ESM.pdf]

Supplemental File
Nature Communications Manuscript NCOMMS-20-39550-T

SUPPLEMENTAL FIGURES AND TABLES

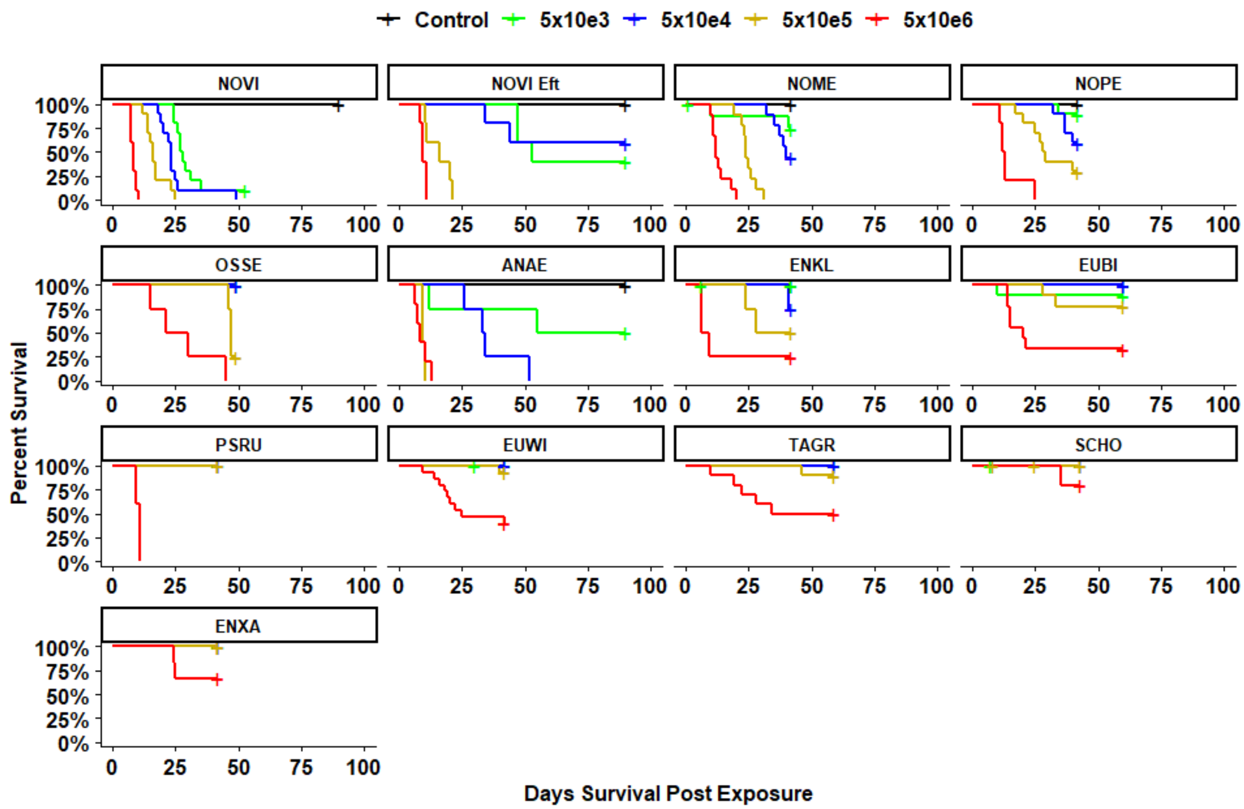

Figure 1. Survival curves among four zoospore exposure doses for species that experienced mortality due to *Batrachochytrium salamandrivorans* (*Bsal*) chytridiomycosis. Curves not shown = 100% survival. ANAE= *Aneides aeneus*, ENKL=*Ensatina eschscholtzii klauberi,* ENXA= *Ensatina eschscholtzii xanthoptica*, EUBI=*Eurycea bislineata,* EUWI=*Eurycea wilderae,* NOME = *Notophthalmus meridionalis,* NOPE=*Notophthalmus perstriatus*, NOVI = *Notophthalmus viridescens,* OSSE=*Osteopilus septentrionalis*, PSRU= *Pseudotriton ruber*, SCHO=*Scaphiopus holbrookii,* and TAGR= *Taricha granulosa*. All species listed here were adults except for NOVI eft = juvenile.

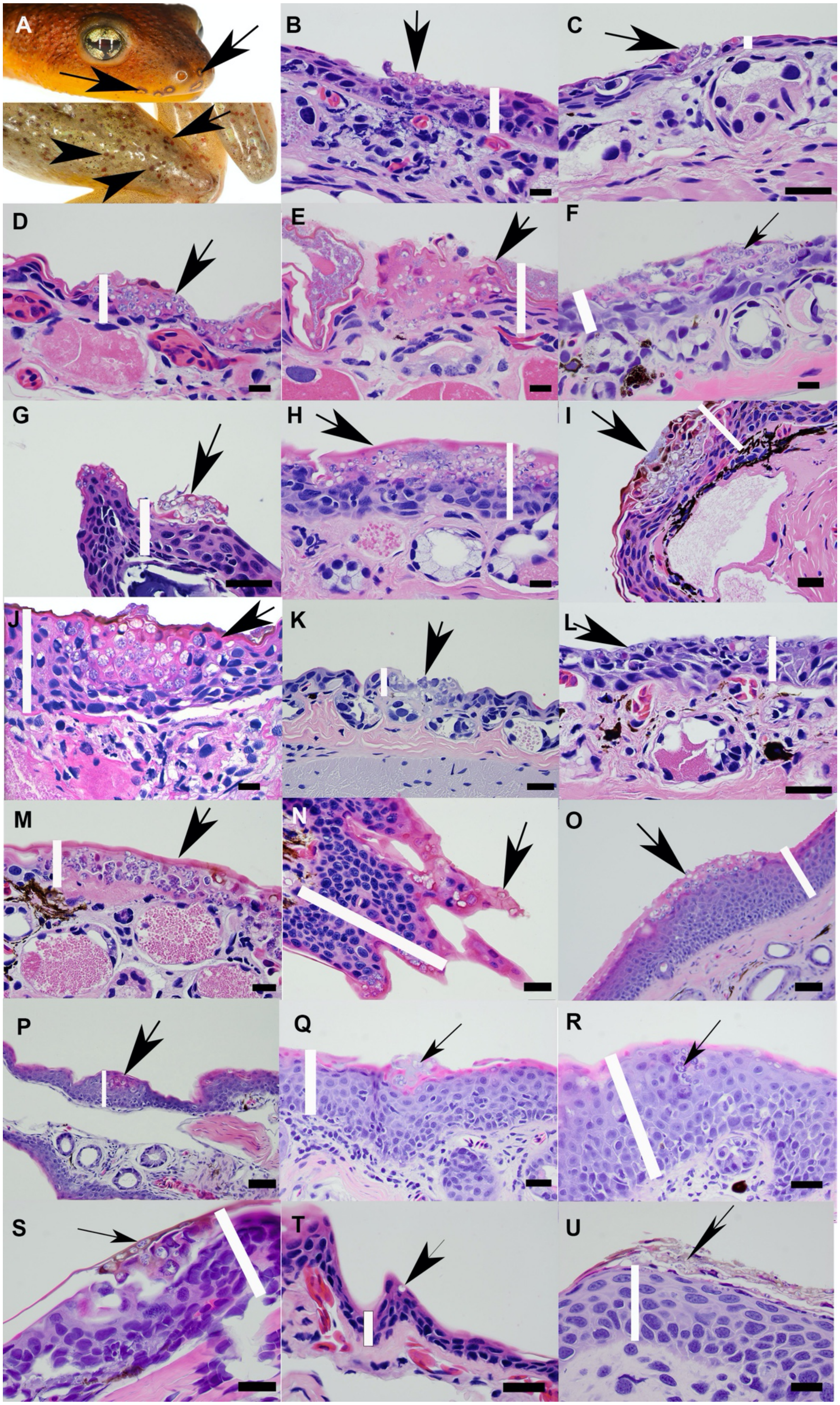
A
B
C
D
E
F
G
H
I
J
K
L
M
N
O
P
Q
R
S
T
U

Figure 2. ***Batrachochytrium salamandrivorans* (*Bsal*) chytridiomycosis in experimentally exposed salamanders and anurans.** Gross lesions included classic target lesions in salamanders (A top arrows) and solid areas of necrosis as well as occasional target lesions in anurans (A bottom arrowheads and arrow, respectively). Hematoxylin and eosin stained skin sections of the gross lesions with epidermal destruction by *Bsal* fungi (arrows) are shown in the following species: adult *Notophthalmus viridescens* (B) juvenile *N. viridescens* (C), *N. perstriatus* (D), *N. meridionalis* (E), adult *Eurycea bislineata* (F), larval *E. bislineata* (G), *E. wilderae* (H), *Taricha granulosa* (I), *T. torosa* (J), *Aneides aeneus* (K), *Pseudotriton ruber* (L), *Ensatina eschollzii klauberi* (M), *E. eschollzii xanthoptica* (N), *Osteopilus septentrionalis* (O)*, and *Scaphiopus holbrookii* (P)*. Species with positive qPCR results that had no obvious gross lesions where *Bsal* was detected using histology included: *Anaxyrus americanus* (Q)*, *Hyla chrysoscelis* (R)*, *Plethodon metcalfi* (S), *Eurycea lucifuga* (T), and *Siren lacertina* (U). At least 5 individuals per species were inspected to verify *Bsal* infection and chytridiomycosis using histology (see Supplemental Table 1). Epidermal layer noted by white bars; * = anurans; all others = salamanders; black scale bars = 20 um.

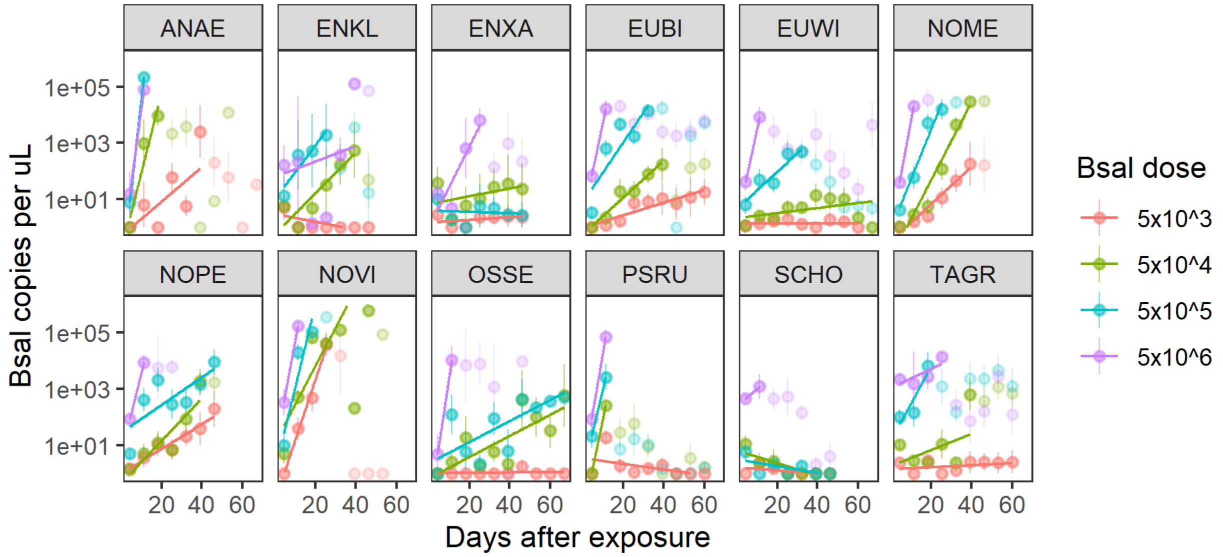

Figure 3. *Bsal* growth rate varies with exposure dose. *Bsal* load (mean and standard error for each sampling period) over the course of the exposure experiments for the species that developed mean loads > 10^3 for at least one of the *Bsal* doses (see Supplementary Text for details). Darker symbols represent the linear portion of the growth curve on a log scale (see Supplementary Text for details), lighter symbols represent data collected after this period. Lines represent linear models fit to the log-transformed raw data for the linear portion of the growth curve. According to the ZINB mixed model described in the text, across all species there was a difference in the rate of *Bsal* increase between treatments (χ^2=33.35, df=6, *P*=9.0 x 10^{-6}). This was largely driven by more rapid development of *Bsal* infection in the 5x10^6 treatment compared to the other treatments – between the first week and second weeks after exposure model-predicted *Bsal* load increased 526% in the 5x10^6 treatment and only 165-320% in the other treatments. All hypothesis tests were two-sided. ANAE= *Aneides aeneus,* ENKL=*Ensatina eschscholtzii klauberi,* ENXA= *Ensatina eschscholtzii xanthoptica*, EUBI=*Eurycea bislineata,* EUWI=*Eurycea wilderae,* NOME = *Notophthalmus meridionalis,* NOPE=*Notophthalmus perstriatus,* NOVI = *Notophthalmus viridescens,* OSSE=*Osteopilus septentrionalis*, PSRU= *Pseudotriton ruber*, SCHO=*Scaphiopus holbrookii,* and TAGR= *Taricha granulosa*.

Table 1. Host susceptibility and conservation risk for 35 North American amphibian species exposed to *Batrachochytrium salamandrivorans* (*Bsal*) at 15°C

Species	Life-stage	% Infection (n)[a]	% Mortality[a]	Avg Copies[a]	Avg Copies SE[a]	Incubation Period[a,b,c]	SE[a,c]	ID-50[c]	SE[c,d]	LD-50[c]	SE[c,d]	Conservation Risk[e]	Gross Lesions[f]	Histology[g]
Notophthalmus viridescens	Adult	100 (10)	100	128072.1	38263	6.4	0.3	2897	Inf	2897	Inf	Very High	Yes	Yes
Notophthalmus viridescens	Juvenile	100(10)	100	304147.9	159247	4.0	0.0	NE	NE	NE	NE	Very High	Yes	Yes
Notophthalmus perstriatus	Adult	100 (10)	100	20067.27	5970	4.0	0.0	NE	NE	71767	1.8	Very High	Yes	Yes
Eurycea bislineata	Adult	100 (9)	66.67	47998.22	6743	4.0	0.0	2987	Inf	2859708	3.0	High	Yes	Yes
Notophthalmus meridionalis	Adult	100 (9)	100	35909.89	10125	4.7	0.7	3708	Inf	20368	1.5	Very High	Yes	Yes
Taricha granulosa	Adult	100 (10)	50	18077.63	4222	4.0	0.0	5925	Inf	4862047	2.0	Low	Yes	Yes
Osteopilus septentrionalis	Adult	100 (4)	100	57135.48	24901	8.5	1.5	6203	Inf	402625	Inf	High	Yes	Yes
Aneides aeneus	Adult	100 (10)	50	35360	14387	7.0	1.0	9528	2.0	1409309	13.6	Moderate	Yes	Yes
Pseudotriton ruber	Adult	100 (5)	100	86275.63	33811	4.0	0.0	15555	1.8	1570681	Inf	Moderate	Yes	Yes
Ensatina eschscholtzii klauberi	Adult	100 (4)	75	29116.23	14397	4.0	0.0	15921	Inf	973838	2.2	Moderate	Yes	Yes
Eurycea wilderae	Adult	100 (15)	66.67	20969.79	4340	5.7	0.8	42452	1.4	2993608	1.4	Low	Yes	Yes
Eurycea lucifuga	Adult	80 (5)	0	164.6402	124	7.0	1.7	105417	2.5	NE	NE	Low	No	Yes
Ensatina e. xanthoptic	Adult	100 (6)	33	44585.16	23441	9.0	2.4	252249	3.5	5871555	Inf	Low	Yes	Yes
Scaphiopus holbrookii	Juvenile	100 (5)	20	1589.924	546	4.0	0.0	412380	2.8	7063138	Inf	Low	No	Yes
Taricha torosa	Adult	60 (10)	0	80		31.0		450990	NE	NE	NE	Low	Yes	Yes
Rana chiricahuensis	Juvenile	62.5 (8)	0	13.17908	6	4.0	0.0	496096	Inf	NE	NE	Low	No	No
Pseudobranchus striatus	Adult	80 (100)	0	122.9917	37	4.0	0.0	1053259	1.9	NE	NE	Low	No	No
Hyla chrysoscelis	Juvenile	50 (10)	0	14.95589	7	6.4	1.5	5000008	1.1	NE	NE	Low	No	Yes
Plethodon shermani	Adult	42.9 (7)	0	7.06142	6	25.0	15.0	5333243	Inf	NE	NE	Low	No	No
Ambystoma opacum	Adult	10 (10)	0	0.134	0	4.0	0.0	8629525	Inf	NE	NE	Low	No	No
Desmognathus ocoee	Adult	30 (10)	0	1.89775	2	6.0	2.0	15859195	4.1	NE	NE	Low	No	No
Notophthalmus viridescens	Larval	17 (12)	0	116		7.0		25648420	Inf	NE	NE	Low	No	No
Ambystoma mexicanum	Adult	50 (10)	0	0.348	0	17.2	3.5	37417161	130.1	NE	NE	Low	No	No
Plethodon metcalfi	Adult	20 (10)	0	1.13275	1	13.0	3.0	51557075	11.4	NE	NE	Low	No	Yes
Siren lacertina	Adult	20 (5)	0	1.313978	1	45.0	0.0	52891331	35.3	NE	NE	Low	No	Yes
Cryptobranchus alleganiensis	Juvenile	1 (7)	0	0.019	0	40.0	0.0	NE	NE	NE	NE	Low	No	No
Anaxyrus americanus	Juvenile	100 (5)	0	552	369	4.0	0.0	NE	NE	NE	NE	Low	No	Yes
Eurycea bislineata	Larval	28 (7)	0	11160.06	2379	NE	NE	NE	NE	NE	NE	Low	No	Yes
Ambystoma maculatum	Juvenile	0 (12)	0	0	0	NE	NE	NE	NE	NE	NE	Low	No	No
Ambystoma laterale	Adult	0 (5)	0	0	0	NE	NE	NE	NE	NE	NE	Resistant	No	No
Ambystoma maculatum	Larval	0 (10)	0	0	0	NE	NE	NE	NE	NE	NE	Resistant	No	No
Desmognathus aeneus	Adult	0 (10)	0	0	0	NE	NE	NE	NE	NE	NE	Resistant	No	No
Desmognathus monticola	Adult	0 (10)	0	0	0	NE	NE	NE	NE	NE	NE	Resistant	No	No
Hemidactylium scutatum	Adult	0 (5)	0	0	0	NE	NE	NE	NE	NE	NE	Resistant	No	No
Necturus maculosus	Adult	0 (5)	0	0	0	NE	NE	NE	NE	NE	NE	Resistant	No	No
Atelopus zeteki	Larval	0 (10)	0	0	0	NE	NE	NE	NE	NE	NE	Resistant	No	No
Rana catesbeiana	Juvenile	0 (6)	0	0	0	NE	NE	NE	NE	NE	NE	Resistant	No	No
Rana catesbeiana	Larval	0 (30)	0	0	0	NE	NE	NE	NE	NE	NE	Resistant	No	No
Rana pipiens	Adult	0 (5)	0	0	0	NE	NE	NE	NE	NE	NE	Resistant	No	No
Rana sylvatica	Juvenile	0 (5)	0	0	0	NE	NE	NE	NE	NE	NE	Resistant	No	No
Rana pipiens	Larval	0 (20)	0	0	0	NE	NE	NE	NE	NE	NE	Resistant	No	No

[a] Highest exposure dose results (see Supplemental Table 2); percent infection and mortality were calculated using the total sample size (n) in this dose; copies refer to *Bsal* fungal DNA copies per microliter.

[b] Incubation period was calculated by averaging the number of days it took for each individual to test positive for *Bsal* DNA using quantitative-PCR for each species.

[c] NE= Not estimable, because either no or all animals became infected or died.

[d] Inf= Infinite standard error estimation due to limits of model estimation based on available data.

[e] Conservation risk (also referred to as mortality risk, Figure 3) was estimated as the product of the median infectious dose (ID-50) and lethal dose (LD-50) estimates, and ranked according to the 25th percentile quartiles. All species that became infected but did not die also were classified as low susceptibility and risk. Species with 100% infection and mortality at all treatment groups were classified as very high conservation risk. Note: NOVI larvae infection only occurred in metamorphosing individuals.

[f] Gross lesions were noted during each observation event. A "yes" in this column indicates that the species had gross lesions typically consistent with *Bsal* chytridiomycosis[1,2]. According to the case definition of *Bsal*, gross signs should not be used to diagnosis *Bsal* chytridiomycosis[1]; histology in combination with another diagnostic methods (e.g., qPCR, culture) are recommended[2].

[g] Histological examinations were performed on each species which had *Bsal* detected via qPCR. A "yes" in this column indicates that *Bsal* infections were confirmed through histological observations (see Supplemental Figure 2).

Table 2. Collecting location and experimental conditions for *Batrachochytrium salamandrivorans* (*Bsal*) exposure experiments. Asterisk (*) indicates collected as eggs from the wild.

Species	Lifestage	Order	Family	IUCN Conservation Status	Source	Location	Start Date (Mo-Yr)	Housing Conditions	Exposure Method	Treatments	n	qPCR Processing Location
Ambystoma laterale	Adult	Caudata	Ambystomatidae	Least Concern	Wild Caught	ME, USA	May-16	Terrestrial	Water Bath	Control	4	UT Knoxville
										5x10e3	5	
										5x10e4	5	
										5x10e5	5	
										5x10e6	5	
Ambystoma maculatum	Larval	Caudata	Ambystomatidae	Least Concern	Wild Caught	MA, USA	Aug-16	Aquatic	Water Bath	Control	.	UMass Boston
										5x10e3	.	
										5x10e4	.	
										5x10e5	.	
										5x10e6	10	
Ambystoma maculatum	Juvenile	Caudata	Ambystomatidae	Least Concern	Wild Caught	MA, USA	Dec-16	Terrestrial	Water Bath	Control	.	UMass Boston
										5x10e3	.	
										5x10e4	.	
										5x10e5	.	
										5x10e6	10	
Ambystoma mexicanum	Adult	Caudata	Ambystomatidae	Critically Endangered	Captive	MA, USA	Apr-17	Aquatic	Water Bath	Control	7	UT Knoxville
										5x10e3	10	
										5x10e4	10	
										5x10e5	10	
										5x10e6	10	
Ambystoma opacum	Adult	Caudata	Ambystomatidae	Least Concern	Wild Caught	TN, USA	Jan-16	Terrestrial	Water Bath	Control	10	UT Knoxville
										5x10e3	10	
										5x10e4	10	
										5x10e5	10	
										5x10e6	10	
Anaxyrus americanus	Juvenile	Anura	Bufonidae	Least Concern	Captive*	TN, USA	Oct-17	Terrestrial	Water Bath	Control	2	UT Knoxville
										5x10e3	.	
										5x10e4	.	
										5x10e5	.	
										5x10e6	5	
Aneides aeneus	Adult	Caudata	Plethodontidae	Near Threatened	Captive	Indoor Ecosystems LLC	Feb-17	Terrestrial	Water Bath	Control	7	UT Knoxville
										5x10e3	10	
										5x10e4	10	
										5x10e5	10	
										5x10e6	10	
Atelopus zeteki	Larval	Anura	Bufonidae	Critically Endangered	Captive	Maryland Zoo	Mar-19	Aquatic	Water Bath	Control	.	UMass Boston
										5x10e3	.	
										5x10e4	.	
										5x10e5	10	
										5x10e6	.	
Cryptobranchus alleganiensis	Juvenile	Caudata	Cryptobranchidae	Near Threatened	Captive	Nashville Zoo	Oct-16	Aquatic	Pipette Exposure	Control	3	UT Knoxville
										5x10e3	6	
										5x10e4	7	
										5x10e5	7	
										5x10e6	7	
Desmognathus aeneus	Adult	Caudata	Plethodontidae	Near Threatened	Captive	Indoor Ecosystems LLC	Jul-16	Terrestrial	Water Bath	Control	5	UT Knoxville
										5x10e3	10	
										5x10e4	10	
										5x10e5	10	
										5x10e6	10	
Desmognathus monticola	Adult	Caudata	Plethodontidae	Least Concern	Wild Caught	GA, USA	Jul-16	Terrestrial	Water Bath	Control	8	UT Knoxville
										5x10e3	10	
										5x10e4	10	
										5x10e5	10	
										5x10e6	10	
Desmognathus ocoee	Adult	Caudata	Plethodontidae	Least Concern	Wild Caught	NC, USA	May-16	Terrestrial	Water Bath	Control	5	UT Knoxville
										5x10e3	10	
										5x10e4	10	
										5x10e5	10	
										5x10e6	10	
Ensatina eschscholtzii klauberi	Adult	Caudata	Plethodontidae	Least Concern	Captive	Indoor Ecosystems LLC	Mar-18	Terrestrial	Water Bath	Control	2	UT Knoxville
										5x10e3	4	
										5x10e4	4	
										5x10e5	4	
										5x10e6	4	

Table 2. Continued.

Species	Lifestage	Order	Family	IUCN Conservation Status	Source	Location	Start Date (Mo-Yr)	Housing Conditions	Exposure Method	Treatments	n	qPCR Processing Location
Ensatina e. xanthoptica	Adult	Caudata	Plethodontidae	Least Concern	Wild Caught	CA, USA	Mar-18	Terrestrial	Water Bath	Control<br>5x10e3<br>5x10e4<br>5x10e5<br>5x10e6	6<br>6<br>6<br>6<br>6	UT Knoxville
Eurycea bislineata	Larval	Caudata	Plethodontidae	Least Concern	Wild Caught	MA, USA	Aug-19<br>Oct-17	Aquatic	Water Bath	Control<br>5x10e3<br>5x10e4<br>5x10e5<br>5x10e6	3<br>5<br>5<br>5<br>7	UTK<br>UMass Boston
Eurycea bislineata	Adult	Caudata	Plethodontidae	Least Concern	Wild Caught	MA, USA	Aug-19	Terrestrial	Water Bath	Control<br>5x10e3<br>5x10e4<br>5x10e5<br>5x10e6	8<br>9<br>9<br>9<br>9	UT Knoxville
Eurycea lucifuga	Adult	Caudata	Plethodontidae	Least Concern	Captive	Indoor Ecosystems LLC	Nov-17	Terrestrial	Water Bath	Control<br>5x10e3<br>5x10e4<br>5x10e5<br>5x10e6	2<br>5<br>5<br>5<br>5	UT Knoxville
Eurycea wilderae	Adult	Caudata	Plethodontidae	Least Concern	Wild / Captive	NC, VA, Indoor Ecosystems LLC	Oct-16<br>Oct-17<br>Nov-17	Terrestrial	Water Bath	Control<br>5x10e3<br>5x10e4<br>5x10e5<br>5x10e6	15<br>11<br>15<br>15<br>15	UT Knoxville
Hemidactylium scutatum	Adult	Caudata	Plethodontidae	Least Concern	Captive	Indoor Ecosystems LLC	Feb-17	Terrestrial	Water Bath	Control<br>5x10e3<br>5x10e4<br>5x10e5<br>5x10e6	5<br>5<br>5<br>5<br>5	UT Knoxville
Hyla chrysoscelis	Adult	Anura	Hylidae	Least Concern	Captive*	TN, USA	May-16	Terrestrial	Water Bath	Control<br>5x10e3<br>5x10e4<br>5x10e5<br>5x10e6	10<br>10<br>10<br>10<br>10	UT Knoxville
Necturus maculosus	Adult	Caudata	Proteidae	Least Concern	Wild Caught	OH, USA	Apr-16	Aquatic	Pipette Exposure	Control<br>5x10e3<br>5x10e4.5<br>5x10e6	2<br>5<br>4<br>5	UT Knoxville
Notophthalmus meridionalis	Adult	Caudata	Salamandridae	Endangered	Captive	Fort Worth Zoo	Apr-17	Terrestrial	Water Bath	Control<br>5x10e3<br>5x10e4<br>5x10e5<br>5x10e6	4<br>9<br>9<br>9<br>9	UT Knoxville
Notophthalmus perstriatus	Adult	Caudata	Salamandridae	Near Threatened	Captive	Omaha Zoo	Apr-17	Terrestrial	Water Bath	Control<br>5x10e3<br>5x10e4<br>5x10e5<br>5x10e6	10<br>10<br>10<br>10<br>10	UT Knoxville
Notophthalmus viridescens	Adult	Caudata	Salamandridae	Least Concern	Wild Caught	TN, USA	Oct-18	Aquatic	Water Bath	Control<br>5x10e3<br>5x10e4<br>5x10e5<br>5x10e6	5<br>10<br>10<br>10<br>10	UT Knoxville
Notophthalmus viridescens	Eft	Caudata	Salamandridae	Least Concern	Wild Caught	PA & MA, USA	Aug-19	Terrestrial	Water Bath	Control<br>5x10e3<br>5x10e4<br>5x10e5<br>5x10e6	5<br>5<br>5<br>5<br>5	UT Knoxville
Notophthalmus viridescens	Larval	Caudata	Salamandridae	Least Concern	Wild Caught	MA, USA	Aug-18	Aquatic	Water Bath	Control<br>5x10e3<br>5x10e4<br>5x10e5<br>5x10e6	12<br>.<br>12<br>12<br>12	UMass Boston
Osteopilus septentrionalis	Adult	Anura	Hylidae	Least Concern	Wild Caught	FL, USA	Jan-19	Terrestrial	Water Bath	Control<br>5x10e3<br>5x10e4<br>5x10e5<br>5x10e6	2<br>4<br>4<br>4<br>4	UT Knoxville

Table 2. Continued.

Species	Lifestage	Order	Family	IUCN Conservation Status	Source	Location	Start Date (Mo-Yr)	Housing Conditions	Exposure Method	Treatments	n	qPCR Processing Location
Plethodon metcalfi	Adult	Caudata	Plethodontidae	Least Concern	Wild Caught	NC, USA	Jul-16	Terrestrial	Water Bath	Control	5	UT Knoxville
										5x10e3	10	
										5x10e4	10	
										5x10e5	10	
										5x10e6	10	
Plethodon shermani	Adult	Caudata	Plethodontidae	Vulnerable	Wild Caught	NC, USA	Jan-16	Terrestrial	Water Bath	Control	7	UT Knoxville
										5x10e3	7	
										5x10e4	7	
										5x10e5	7	
										5x10e6	7	
Pseudobranchus striatus	Adult	Caudata	Sirenidae	Least Concern	Wild Caught	FL, USA	Jun-19	Aquatic	Water Bath	Control	5	UT Knoxville
										5x10e3	5	
										5x10e4	5	
										5x10e5	5	
										5x10e6	5	
Pseudotriton ruber	Adult	Caudata	Plethodontidae	Least Concern	Captive	Indoor Ecosystems LLC	Nov-17	Terrestrial	Water Bath	Control	5	UT Knoxville
										5x10e3	5	
										5x10e4	5	
										5x10e5	5	
										5x10e6	5	
Rana catesbeiana	Juvenile	Anura	Ranidae	Least Concern	Wild Caught	NC,USA	Mar-16	Aquatic	Water Bath	Control	6	UT Knoxville
										5x10e3	6	
										5x10e4	6	
										5x10e5	6	
										5x10e6	6	
Rana catesbeiana	Larval	Anura	Ranidae	Least Concern	Captive*	TN, USA	Nov-18	Terrestrial	Water Bath	Control	10	UMass Boston
										5x10e3	.	
										5x10e4	10	
										5x10e5	10	
										5x10e6	30	
Rana chiricahuensis	Juvenile	Anura	Ranidae	Vulnerable	Captive *	Fort Worth Zoo	Mar-16	Terrestrial	Water Bath	Control	8	UT Knoxville
										5x10e3	8	
										5x10e4	8	
										5x10e5	8	
										5x10e6	8	
Rana pipiens	Larval	Anura	Ranidae	Least Concern	Captive*	UMass Boston	Jul-19	Aquatic	Water Bath	Control	15	UMass Boston
										5x10e3	15	
										5x10e4	15	
										5x10e5	15	
										5x10e6	5	
Rana pipiens	Juvenile	Anura	Ranidae	Least Concern	Captive *	UMass Boston	Aug-19	Terrestrial	Water Bath	Control	.	UMass Boston
										5x10e3	.	
										5x10e4	.	
										5x10e5	.	
										5x10e6	5	
Rana sylvatica	Juvenile	Anura	Ranidae	Least Concern	Captive*	TN, USA	Mar-16	Terrestrial	Water Bath	Control	5	UT Knoxville
										5x10e3	5	
										5x10e4	5	
										5x10e5	5	
										5x10e6	5	
Scaphiopus holbrookii	Juvenile	Anura	Scaphiopodidae	Least Concern	Captive*	TN, USA	Oct-17	Terrestrial	Water Bath	Control	5	UT Knoxville
										5x10e3	5	
										5x10e4	5	
										5x10e5	5	
										5x10e6	5	
Siren lacertina	Adult	Caudata	Sirenidae	Least Concern	Wild Caught	FL, USA	Jun-19	Aquatic	Pipette Exposure	Control	5	UT Knoxville
										5x10e3	5	
										5x10e4	5	
										5x10e5	5	
										5x10e6	5	
Taricha granulosa	Adult	Caudata	Salamandridae	Least Concern	Wild Caught	CA, USA	Sep-17	Terrestrial	Water Bath	Control	8	UT Knoxville
										5x10e3	10	
										5x10e4	10	
										5x10e5	10	
										5x10e6	10	
Taricha torosa	Adult	Caudata	Salamandridae	Least Concern	Wild Caught	CA, USA	Mar-18	Terrestrial	Water Bath	Control	10	Washington State University
										1x10e3	10	
										1x10e4	10	
										1x10e5	10	
										1x10e6	10	

Table 3. Parameter estimates for analysis of *Bsal* growth rate across species.

Random effects

Group	Negative binomial<br>Standard deviation	Correlation	Zero-inflated<br>Standard deviation	Correlation
Species (intercept)	1.94		4.25	
Species (slope – Day of swab)	0.01	-0.36	0.05	-0.88
Individual (intercept)	2.73		2.18	

Fixed effects

Parameter	Negative binomial	Zero-inflated
(Intercept)	2.07	1.92
Day_of_Swab	0.12	-0.05
Treatment5x10^4	0.72	-1.14
Treatment5x10^5	4.27	-3.12
Treatment5x10^6	5.29	0.34
Day_of_Swab:Treatment5x10^4	0.06	-0.05
Day_of_Swab:Treatment5x10^5	0.04	-0.06
Day_of_Swab:Treatment5x10^6	0.17	-1.19

Table 4. Post-hoc tests for differences in *Bsal* growth rate between exposure doses across species. All tests two-sided with Tukey adjustments for multiple comparisons.

	Negative binomial			Zero-inflated		
**Contrast**	***t***	**df**	***P***	***t***	**df**	***P***
10^3 - 10^4	-2.71	1986	0.035	2.53	1986	0.055
10^3 - 10^5	-1.56	1986	0.40	2.40	1986	0.078
10^3 - 10^6	-3.15	1986	0.0091	2.35	1986	0.088
10^4 - 10^5	1.18	1986	0.64	0.63	1986	0.92
10^4 - 10^6	-2.08	1986	0.16	2.26	1986	0.11
10^5 - 10^6	-2.47	1986	0.065	2.22	1986	0.11

SUPPLEMENTAL NOTE 1

R Code for Bsal Detection Estimates

```
##### Calculation 1 ########

N = 1283 # Total sample
n = 336 # Control subset
true_prevalence = 0.01 # True prevalence/probability of being infected

# What is the probability of not detecting Bsal in 336 tested individuals.  Assuming all individuals
# have an equal probability of having Bsal
prob_of_detection = 1 - (1 - true_prevalence)^n

# With true_prevalence = 0.05, the probability of detecting Bsal approx 1 -> you are going to detect it on
one of your control animals
# With true_prevalence = 0.01, the probability of detection Bsal = 0.966 -> There is a < 5% change that
you falsely say no Bsal on controls

# Check with a simulation
sims = 10000
detected = array(NA, sims)

for(i in 1:sims){
	newts = rbinom(N, 1, true_prevalence)
	samp_newts = any(sample(newts, n, replace=FALSE))
	detected[i] = samp_newts
}

#Probability of detecting Bsal
sum(detected) / sims

# Same results as above: < 5% chance of failing to detect Bsal if it exists.

##### Calculation 2 ########

# Let's now say each individual has a different probability of having Bsal, but that the
# average is 0.01 or 0.05
true_prevalence = 0.01
phi = 1 # Dispersion parameter of beta distribution
alpha = phi * true_prevalence
beta = phi*(1 - true_prevalence)

# Check with a simulation
sims = 10000
detected = array(NA, sims)

for(i in 1:sims){
```

```
	# Beta binomial simulation
	probs = rbeta(N, alpha, beta)
	newts = rbinom(N, size=1, prob=probs)
	samp_newts = any(sample(newts, n, replace=FALSE))
	detected[i] = samp_newts
}

# Probabiliy of detecting Bsal
sum(detected) / sims

# There is no substantial change -> there is still less than a 5% that you fail to detect
# Bsal given its presence.
```

SUPPLEMENTAL INFORMATION REFERENCES

1 White, C. L. *et al.* Amphibian: A case definition for Batrachochytrium salamandrivorans chytridiomycosis. *Herpetological Review* **47**, 207-207 (2016).

2 Thomas, V. *et al.* Recommendations on diagnostic tools for Batrachochytrium salamandrivorans. *Transbound Emerg Dis* **65**, e478-e488, doi:10.1111/tbed.12787 (2018).
