# Supplementary material for: Broad host susceptibility of North American amphibian species to Batrachochytrium salamandrivorans suggests high invasion potential and biodiversity risk
Source: Nat Commun. 2023 Jun 5;14:3270. doi: 10.1038/s41467-023-38979-4 (PMC10241899; doi:10.1038/s41467-023-38979-4)
Supplement: Supplementary file 2 — Reporting Summary [file 41467_2023_38979_MOESM2_ESM.pdf]

nature research

Corresponding author(s): Matthew J. Gray

Last updated by author(s): May 4, 2023

Reporting Summary

Nature Research wishes to improve the reproducibility of the work that we publish. This form provides structure for consistency and transparency in reporting. For further information on Nature Research policies, see our Editorial Policies and the Editorial Policy Checklist.

Statistics

For all statistical analyses, confirm that the following items are present in the figure legend, table legend, main text, or Methods section.

n/a	Confirmed	
☐	☒	The exact sample size (*n*) for each experimental group/condition, given as a discrete number and unit of measurement
☐	☒	A statement on whether measurements were taken from distinct samples or whether the same sample was measured repeatedly
☐	☒	The statistical test(s) used AND whether they are one- or two-sided<br>*Only common tests should be described solely by name; describe more complex techniques in the Methods section.*
☒	☐	A description of all covariates tested
☐	☒	A description of any assumptions or corrections, such as tests of normality and adjustment for multiple comparisons
☐	☒	A full description of the statistical parameters including central tendency (e.g. means) or other basic estimates (e.g. regression coefficient) AND variation (e.g. standard deviation) or associated estimates of uncertainty (e.g. confidence intervals)
☐	☒	For null hypothesis testing, the test statistic (e.g. *F*, *t*, *r*) with confidence intervals, effect sizes, degrees of freedom and *P* value noted<br>*Give P values as exact values whenever suitable.*
☒	☐	For Bayesian analysis, information on the choice of priors and Markov chain Monte Carlo settings
☒	☐	For hierarchical and complex designs, identification of the appropriate level for tests and full reporting of outcomes
☐	☒	Estimates of effect sizes (e.g. Cohen's *d*, Pearson's *r*), indicating how they were calculated

Our web collection on statistics for biologists contains articles on many of the points above.

Software and code

Policy information about availability of computer code

Data collection	All data used for our analyses are available at the following public repository: https://doi.org/10.7290/pJ8IWH7DuE . We generated all data for our analyses from the experiments we performed, except for species distributions needed for the geographic risk analyses. To estimate the geographic range of each salamander species, we extracted species polygons for the 168 salamander species from the IUCN species distribution database (https://www.iucnredlist.org).
Data analysis	Bsal Loads: To estimate Bsal growth, we fit our models using the glmmTMB package in R; conformity with model assumptions was evaluated using the DHARMa package. Post-hoc tests evaluating the differences in slopes between doses were conducted separately for the zero-inflated and negative binomial portions of the model using t-tests with Tukey adjustments for multiple comparisons; these post-hoc tests were performed using functions in the emmeans package in R.<br><br>ID- and LD-50: For species or age classes that became infected or died, we estimated median infectious and lethal doses (ID-50 and LD-50 values, respectively) using log-probit analyses in the MASS package in R.<br><br>Phylogenetic Signal: To determine whether Bsal susceptibility followed a phylogenetic signal, we constructed a phylogenetic tree for the adult amphibians tested using TimeTree, and estimated Blomberg's K using the "Kcalc" function from the "picante" package in R.<br><br>Risk Maps: To estimate the geographic range of each salamander species, we extracted species polygons for the 168 salamander species with known phylogenetic relationships from the IUCN species distribution database (iucnredlist.org). We used the geographic distribution polygons to generate county-level occurrences for each species using the clip function found in the ArcMap (v.10.7) analysis toolbox.

For manuscripts utilizing custom algorithms or software that are central to the research but not yet described in published literature, software must be made available to editors and reviewers. We strongly encourage code deposition in a community repository (e.g. GitHub). See the Nature Research guidelines for submitting code & software for further information.

Data

Policy information about availability of data

All manuscripts must include a data availability statement. This statement should provide the following information, where applicable:

- Accession codes, unique identifiers, or web links for publicly available datasets
- A list of figures that have associated raw data
- A description of any restrictions on data availability

All raw data used for our analyses are available at the following public repository: https://doi.org/10.7290/pJ8IWH7DuE. Summarized data are provided in Figures 1-3 and in the Supplemental Information file. There are no restrictions on use.

Field-specific reporting

Please select the one below that is the best fit for your research. If you are not sure, read the appropriate sections before making your selection.

☐ Life sciences ☐ Behavioural & social sciences ☒ Ecological, evolutionary & environmental sciences

For a reference copy of the document with all sections, see nature.com/documents/nr-reporting-summary-flat.pdf

Ecological, evolutionary & environmental sciences study design

All studies must disclose on these points even when the disclosure is negative.

Study description	This study involved estimating the susceptibility of 35 amphibian species to the fungal pathogen, Batrachochytrium salamandrivorans (Bsal). Amphibians were acquired from captive collections or the wild and transported overnight (<24 hrs) in individual containers with moistened paper towels (if a terrestrial species) or water (if an aquatic species) to the University of Tennessee, Washington State University or University of Massachusetts-Boston. Our experiments were conducted in biosecure environmental chambers set at 15 C with a 12:12 light:dark cycle. Our treatment (factor) was Bsal zoospore exposure dose, with a target of four doses per species: 5 x 10e3, 10e4, 10e5, 10e6. We attempted to test 10 individuals per dose, which is sufficient to estimate infectious dose (ID)- and lethal dose (LD)-50 concentrations, and were used as metric of host susceptibility. Sample size per dose and number of zoospore doses varied among species, depending on availability of animals for the experiments. Final sample sizes and number of doses are presented in Supplemental Table 2.
Research sample	Thirty-five amphibian species, which are listed in Supplemental Tables 1 and 2. Because sex could not be reliably determined phenotypically for most of our species, it was not considered in the experimental design.
Sampling strategy	Amphibians were opportunistically acquired. We attempted to test 10 individuals per dose per species, which is sufficient to estimate ID- and LD-50 concentrations. Sample size per dose and number of zoospore doses varied among species, depending on availability of animals for the experiments. Final sample sizes and number of doses are presented in Supplemental Table 2.
Data collection	Survival of amphibians was monitored twice daily and individuals that displayed loss of righting reflex were humanely euthanized using benzocaine hydrochloride. Bsal growth on salamanders was measured every six days by swabbing the skin of each animal, extracting gDNA, and performing qPCR following standardized protocols (see Methods). Data that were analyzed included survival, infection, and Bsal load on the skin of animals. Histological evidence of Bsal chytridiomycosis also is provided; see Supplemental Figure 2 and Supplemental Table 2. Data were collected by Davis Carter, Patrick Cusaac, Anna Peterson, Ross Whetstone, Andreas Hertz, Aura Muniz-Torres, Molly Bletz, John Romansic, Wesley Sheley, Allan Pessier, and Debra Miller.
Timing and spatial scale	Our experiments were performed between January 2016 and August 2019 (see Supplemental Table 2).
Data exclusions	We excluded two species (Aquiloeurycea, Chiropterotriton) from the manuscript that tested positive for Batrachochytrium dendrobatidis (Bd) as per a reviewer's request, because of possible confounding factors associated with Bd and Bsal co-infection. Only 12 species were used for the Bsal load analyses because insufficient infection data existed to fit models for the other species.
Reproducibility	We did not repeat our experiments.
Randomization	All animals were randomly assigned to zoospore doses, including controls.
Blinding	Blinding of infection and mortality data was not possible, because the individuals that collected these data also contributed to the analyses. Also, the pathologists (Miller and Pessier) were not blinded to the qPCR data because their goal was to determine anatomically whether infected individuals developed Bsal chytridiomycosis.

Did the study involve field work? ☐ Yes ☒ No

Reporting for specific materials, systems and methods

We require information from authors about some types of materials, experimental systems and methods used in many studies. Here, indicate whether each material, system or method listed is relevant to your study. If you are not sure if a list item applies to your research, read the appropriate section before selecting a response.

Materials & experimental systems

n/a	Involved in the study
☒	☐ Antibodies
☒	☐ Eukaryotic cell lines
☒	☐ Palaeontology and archaeology
☐	☒ Animals and other organisms
☒	☐ Human research participants
☒	☐ Clinical data
☒	☐ Dual use research of concern

Methods

n/a	Involved in the study
☒	☐ ChIP-seq
☒	☐ Flow cytometry
☒	☐ MRI-based neuroimaging

Animals and other organisms

Policy information about studies involving animals; ARRIVE guidelines recommended for reporting animal research

Laboratory animals	The 35 species that we tested are listed in Supplemental Table 1. All animals were adults, except where noted in Supplemental Tables 1 and 2.
Wild animals	For species captured from the wild, they were transported by vehicle in individual containers to laboratories (in <12 hrs) at the University of Tennessee, Washington State University or University of Massachusetts-Boston. All animals were humanely euthanized at the end of the experiments and preserved in 10% formalin for histological analyses.
Field-collected samples	In the laboratory, terrestrial amphibians were held in 710-mL plastic containers with a moist paper towel and plastic, opaque cover object to reduce stress. Fully aquatic amphibians or larvae were held in either 20-L glass aquaria (large aquatic [e.g., Siren] or stream-dwelling [Cryptobranchus] species) or in 1 – 3-L tubs containing 500 mL of dechlorinated water and a plastic cover object, depending on body size (see Supplemental Table 2). Animals were fed a diet of either brine shrimp, alfalfa pellets, blood worms, bean beetles, fruit flies or crickets every three days depending on their age class, body size and whether they were aquatic or terrestrial. Animals were acclimated in the laboratory approximately two weeks before experiments began. Containers were maintained between 20 – 22°C until one week prior to exposure trials, after which they were placed in Conviron® environmental chambers (Winnipeg, Canada) or temperature-controlled rooms. The temperature was decreased by 3°C per day until reaching the target temperature of 15°C, which has been the standard for Bsal experiments testing host susceptibility. The ambient light:dark cycle prior to and during experiments was 12:12 hours, respectively.
Ethics oversight	All procedures followed husbandry and euthanasia recommendations provided by the American Veterinary Medical Association and the Association of Zoos and Aquariums. All animal procedures were approved under Institutional Animal Care and Use Committee (IACUC) protocols 2395, 2014003, and 4749 at the University of Tennessee, University of Massachusetts-Boston and Washington State University-Vancouver, respectively. IACUC approval for the Atelopus zeteki experiment was obtained from the Maryland Zoo.

Note that full information on the approval of the study protocol must also be provided in the manuscript.
